# Low-Density Lipoprotein Cholesterol and Mortality in Peritoneal Dialysis

**DOI:** 10.3389/fnut.2022.910348

**Published:** 2022-07-21

**Authors:** Xianfeng Wu, Lei Zhou, Xiaojiang Zhan, Yueqiang Wen, Xiaoyang Wang, Xiaoran Feng, Niansong Wang, Fenfen Peng, Junnan Wu

**Affiliations:** ^1^Department of Nephrology, Shanghai Jiao Tong University Affiliated Sixth People’s Hospital, Shanghai, China; ^2^Clinical Research Center for Chronic Kidney Disease, Shanghai Jiao Tong University Affiliated Sixth People’s Hospital, Shanghai, China; ^3^Evergreen Tree Nephrology Association, Guangzhou, China; ^4^Department of Nephrology, The First Affiliated Hospital of Nanchang University, Nanchang, China; ^5^Department of Nephrology, The Second Affiliated Hospital of Guangzhou Medical University, Guangzhou, China; ^6^Department of Nephrology, The First Affiliated Hospital of Zhengzhou University, Zhengzhou, China; ^7^Department of Nephrology, Jiujiang No. 1 People’s Hospital, Jiujiang, China; ^8^Department of Nephrology, Zhujiang Hospital of Southern Medical University, Guangzhou, China; ^9^Department of Nephrology, Zhejiang University Medical College Affiliated Sir Run Run Shaw Hospital, Hangzhou, China

**Keywords:** peritoneal dialysis, mortality, low-density lipoprotein cholesterol (LDL-C), nutrition–clinical, cardiovascular mortality

## Abstract

**Background:**

In dialysis patients, lowering low-density lipoprotein cholesterol (LDL-C) did not provide benefits, which seemed implausible in clinical practice. We hypothesized a U-shaped association between LDL-C and mortality in dialysis patients.

**Methods:**

In this multi-center retrospective real-world cohort study, 3,565 incident Chinese peritoneal dialysis (PD) patients between January 1, 2005, and May 31, 2020, were included. The associations between baseline LDL-C and mortality were examined using cause-specific hazard models.

**Results:**

Of 3,565 patients, 820 died, including 415 cardiovascular deaths. As compared with the reference range (2.26-2.60 mmol/L), both higher levels of LDL-C (> 2.60 mmol/L) and lower levels of LDL-C (< 2.26 mmol/L) were associated with increased risks of all-cause mortality (hazard ratio [HR],1.35, 95% confidence index [CI], 1.09-1.66; HR 1.36, 95%CI, 1.13-1.64) and cardiovascular mortality (HR, 1.31, 95% CI, 1.10-1.72; HR, 1.64; 95% CI, 1.22-2.19). Malnutrition (albumin < 36.0 g/L) modified the association between LDL-C and cardiovascular mortality (P for interaction = 0.01). A significantly increased risk of cardiovascular mortality was observed among patients with malnutrition and lower levels of LDL-C (HR 2.96, 95%CI 1.43-6.12) or higher levels of LDL-C (HR 2.81, 95%CI 1.38-5.72).

**Conclusion:**

Low and high levels of LDL-C at the start of PD procedure were associated with increased all-cause and cardiovascular mortality risks. Malnutrition may modify the association of LDL-C with cardiovascular mortality.

## Introduction

Low-density lipoprotein cholesterol (LDL-C) is a well-established causal risk factor for the development of cardiovascular disease (1). Many prospective randomized controlled trials of lipid-lowering drug therapy clearly show that lowering LDL-C levels reduces the risk of future cardiovascular events (2–4). Interestingly, a prospective cohort study from Denmark reported that in the general population, the association between levels of LDL-C and the risk of all-cause mortality was U-shaped, with low and high levels associated with an increased risk of all-cause mortality (5). A recent study of young Koreans not taking lipid-lowering drugs showed a U-shaped association between LDL-C levels and mortality (6). Apparently, a U-shaped association seems more plausible in clinical practice.

In dialysis patients, lowering LDL-C did not provide benefits. Two large, well-designed trials examined the effect of statin therapy on the combined endpoint of death from cardiovascular causes, non-fatal myocardial infarction, and stroke in dialysis patients (7, 8). Despite a significant decrease in serum LDL-C levels, both trials found that the initiation of statin therapy provided no cardiovascular benefit in these populations. Another multi-center, prospective cohort study of 630 incident Korean peritoneal dialysis (PD) patients reported that total cholesterol, high-density lipoprotein cholesterol (HDL-C), and LDL-C were also not associated with mortality (9). Another study showed that a combination of statin and ezetimibe benefits the prognosis in non-dialysis chronic kidney disease and dialysis patients. The beneficial effect on atherosclerotic events was not statistically significant in the dialysis subgroup (10). Furthermore, the KDIGO guidelines recommend that statin therapy not be routinely initiated in dialysis patients (11). It is worth noting that these findings above may be implausible in clinical practice. We hypothesized that a U-shaped association between LDL-C and mortality, and there may be an optimal range of LDL-C, which was associated with the lowest mortality risk in dialysis patients. The two large well-designed trials above may lower levels of LDL-C less than the optimal range, which may contribute to a high risk of mortality. Thus, we conducted a real-world study to examine the association between LDL-C and mortality in continuous ambulatory peritoneal dialysis (CAPD) patients.

## Materials and Methods

### Study Design and Participants

We conducted a retrospective real-world cohort study that included 3,565 incident Chinese CAPD patients from five PD centers in China between January 1, 2005, and May 31, 2020. No patients were excluded in the primary analysis to evaluate the association between LDL-C one week before the start of PD and mortality in the real-world setting. The data were anonymous, and the need for informed consent was waived. The study protocol complied with the Declaration of Helsinki and had full approval from each Clinical Research Ethics Committee.

### Data Collection and Follow up

Two well-trained nurses collected demographic data, comorbidities, and laboratory data one week (5.3 ± 1.2 days) before the start of PD in each facility, including age at study entry, sex, body mass index, current smoker, current alcohol use, systolic blood pressure, comorbidities (diabetes mellitus, prior cardiovascular disease, and hypertension), medication use (beta-blockers, angiotensin-converting enzyme inhibitors/angiotensin II receptor blockers [ACEI/ARBs], diuretics, and statins), and laboratory measurements (serum albumin, estimated glomerular filtration rate [eGFR], total cholesterol, HDL-C, and LDL-C).

The primary outcome was all-cause and cardiovascular mortality. Details for the CAPD follow-up were previously described elsewhere (12). The follow-up period was from the start of PD to the date of death, transfer to hemodialysis, receiving renal transplantation, transfer to other dialysis centers, loss of follow-up, or May 31, 2020. Patients who were lost to follow-up were censored at the date of the last examination (Supplementary Materials).

### Statistical Analysis

Continuous variables were presented as means with standard deviations (SDs) for normally distributed data or medians with interquartile range (IQR) for skewed data. The normality of the parameters was examined using the Shapiro–Wilk test. Categorical variables were expressed as the number of patients. We used restricted-cubic-spline plots to explore the shape of the association between LDL-C and mortality, fitting a restricted-cubic-spline function with four knots (at the 25th, 50th, 75th, and 95th percentiles) (13). All parameters were compared among groups based on restricted cubic spline plots for the primary analysis.

To explore the association of LDL-C with mortality, we primarily used cause-specific hazard models. We then constructed sub-distribution hazard models to confirm the association observed in the primary analysis. Transfer to hemodialysis, receiving renal transplantation, transfer to other centers, and loss of follow-up before death were considered competing risks. The main difference between the two hazard models is that subjects experiencing a competing risk event remain in the risk set in the subdistribution hazard model but are removed in the cause-specific hazard model (14, 15). These models were constructed after the adjustment of the following variables. The univariate model represented unadjusted hazard ratios (HRs). The multivariate model was adjusted for age, sex, body mass index, current smoker, current alcohol use, comorbidities, medication use, and laboratory measurements (excluded LDL-C). The results from multivariable hazard models were presented as HRs and 95% confidence intervals (95% CIs). Cumulative primary outcomes were derived using the cumulative incidence function for a competing risk, and the difference among curves was analyzed using the Gray test. To evaluate the modification effects of subgroups on the association between LDL-C and mortality, we tested for interactions of age, sex, diabetes mellitus, prior cardiovascular disease, hypertension, and malnutrition (defined as serum albumin levels < 36.0 g/L) (16).

To minimize the potential for reverse causation, we conducted analyses that excluded patients with prior cardiovascular disease or those deaths in the first two years of follow-up. In addition, as for those patients with a short-term follow-up period, the interesting outcomes may not be wholly observed with under-reporting of the incidence of mortality. We further analyzed the association in those patients with at least 24 months of follow-up for fully observing outcomes. We also examined the association in patients with age ≥ 18 years, those with a follow-up period ≥ three months, and those without statin use. Missing data for low-density lipoprotein (n = 39) or any other explanatory variables (n = 147) at the start of PD were replaced by the most recent available values by checking patients’ medical records of receiving the first PD procedure. All analyses were conducted with Stata 15.1. statistical software (StataCorp, College Station, TX).

## Results

### Baseline Characteristics

**FIGURE 1:**
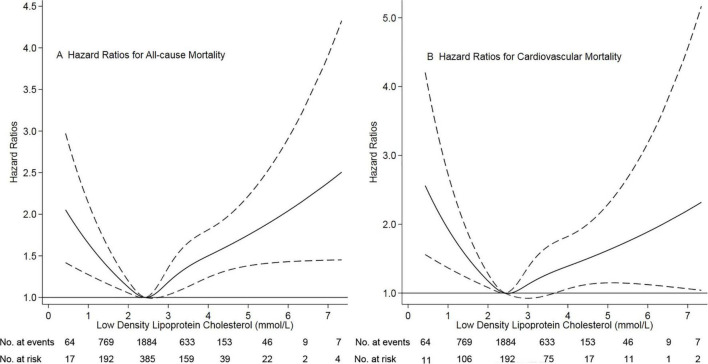
Association of low-density lipoprotein cholesterol with risk of mortality. Panel **(A)** showed a restricted-cubic-spline plot of the association between LDL-C and all-cause mortality. Panel **(B)** showed a restricted cubic spline plot of the association between LDL-C and cardiovascular mortality. All plots were adjusted for age, sex, body mass index, current smoker, current alcohol use, systolic blood pressure, comorbidities, medication use, and laboratory variables. Dashed lines indicate 95% confidence intervals. The median LDL-C (2.43 mmol/L, [89 mg/dL]) was the reference standard, indicated by the grayline. LDL-C, low-density lipoprotein cholesterol.

Of 3,565 patients, the mean age was 49.2 ± 15.1 years (range, 3 to 101 years), 52.1% of patients were male sex. The mean LDL-C levels were 2.56 ± 0.89 mmol/L (range 0.4 to 9.4 mmol/L). We chose the reference group based on the results of restricted-cubic-spline analysis, in which hazard ratios were 1.0 compared with the median LDL-C (2.43 mmol/L). Based on restricted cubic spline plots for the primary outcome, we selected a level of 2.26 to 2.60 mmol/L (87 to 101 mg/dL) as the reference category for LDL-C (Figure 1). There were 1,225 (34.4%) patients with LDL levels < 2.26 mmol/L and 1,439 (40.3%) with LDL levels > 2.60 mmol/L. Thus, 2664 (74.7%) patients were at higher risk of mortality. Table 1 presented the characteristics of patients by categories of baseline LDL-C. Baseline variables were markedly different among the low, moderate, and high groups. The high group had higher systolic blood pressure and total cholesterol levels, frequency of diabetes mellitus, prior cardiovascular disease, and hypertension. In contrast, the low group had lower systolic blood pressure levels, a lower frequency of diabetes mellitus, and prior cardiovascular disease.

**TABLE 1:** Baseline patient characteristics by categories of low-density lipoprotein cholesterol.

		Low-density lipoprotein cholesterol			
	Overall	Low (< 2.26 mmol/L)	Moderate (2.26-2.60 mmol/L)	High (> 2.60 mmol/L)	P-value
Number of patients, *n*	3565	1225	901	1439	
LDL-C (mmol/L)	2.56 ± 0.89	2.41 ± 0.09	1.73 ± 0.40	3.36 ± 0.76	
Age (years)	49.2 ± 15.1	49.1 ± 15.2	48.9 ± 15.2	49.4 ± 15.0	0.729
Male sex, n (%)	1856 (52.1%)	657 (53.6%)	495 (54.9%)	704 (48.9%)	0.007
Body mass index (kg/m^2^)	22.3 ± 3.3	22.1 ± 3.2	22.0 ± 3.0	22.6 ± 3.5	< 0.001
Systolic blood pressure (mmHg)	137.3 ± 22.8	133.3 ± 21.6	137.6 ± 22.1	140.6 ± 23.7	< 0.001
Current smoker, *n* (%)	354 (9.9%)	128 (10.4%)	63 (7.0%)	163 (11.3%)	0.002
Current alcohol use, *n* (%)	129 (3.6%)	45 (3.7%)	21 (2.3%)	63 (4.4%)	0.036
**Comorbidities, *n* (%)**					
Diabetes mellitus	674 (18.9%)	169 (13.8%)	140 (15.5%)	365 (25.4%)	< 0.001
Prior cardiovascular disease	379 (10.6%)	82 (6.7%)	85 (9.4%)	212 (14.7%)	< 0.001
Hypertension	2469 (69.3%)	791 (64.6%)	573 (63.6%)	1105 (76.8%)	< 0.001
**Medication use, *n* (%)**					
Beta-blocker	1338 (37.5%)	394 (32.2%)	304 (33.7%)	640 (44.5%)	< 0.001
Diuretics	557 (15.6%)	145 (11.8%)	121 (13.4%)	291 (20.2%)	< 0.001
Statin	524 (14.7%)	156 (12.7%)	100 (11.1%)	268 (18.6%)	< 0.001
**Laboratory measurements**					
Albumin (g/L)	34.5 ± 5.3	34.5 ± 5.0	35.0 ± 5.3	34.1 ± 5.5	< 0.001
eGFR (mL/min*1.73m^2^)	7.19 ± 3.83	7.20 ± 3.45	6.82 ± 3.73	7.50 ± 4.11	< 0.001
Total Cholesterol (mmol/L)	4.38 ± 1.19	4.72 ± 0.32	3.38 ± 0.58	5.01 ± 1.35	< 0.001
HDL (mmol/L)	1.14 ± 0.40	1.18 ± 0.36	1.03 ± 0.32	1.20 ± 0.45	< 0.001

*LDL-C, low-density lipoprotein cholesterol; eGFR, estimated glomerular filtration rate; HDL-C, high-density lipoprotein cholesterol.*

### LDL-C and Outcomes

**FIGURE 2:**
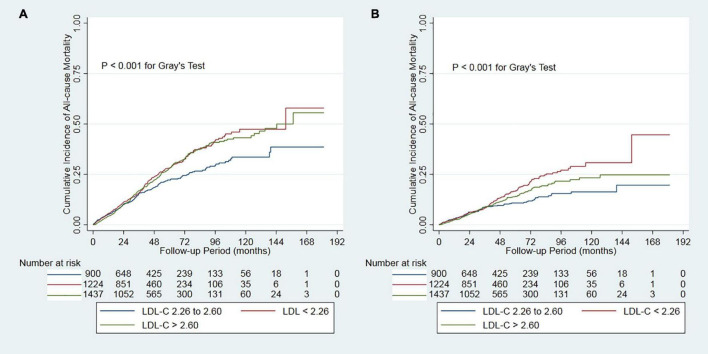
Cumulative mortality by categories of low-density lipoprotein cholesterol. Panel **(A)** showed cumulative all-cause mortality by categories of LDL-C. Panel **(B)** showed cumulative cardiovascular mortality by categories of LDL-C. LDL-C, low-density lipoprotein cholesterol.

During the 14131.6 person-years of follow-up, 820 (23.0%) patients died, 481 (13.5%) patients transferred to hemodialysis, 241 (6.8%) patients received renal transplantation, 459 (12.9%) patients transferred to other dialysis centers, and 61 (1.7%) patients had been the loss of follow-up. Of 820 deaths, 415 (50.6%) deaths were due to cardiovascular disease, 142 (17.3%) deaths due to infectious disease, 76 (9.3%) deaths due to gastrointestinal bleeding, 15 (1.8%) deaths due to malignancy, 73 (8.9%) deaths due to other reasons, and 99 (12.1%) deaths due to unknown reasons. Deaths occurred in 292 (63.8/1000 person-years), 181 (46.5/1000 person-years), and 347 (61.3/1000 person-years) patients in those < 2.26, 2.26-2.60, and > 2.60 mmol/L patients, respectively (Table 2). Cumulative all-cause mortality and cardiovascular mortality were significantly lowest in the moderate group (P < 0.001, Figure 2). The adjusted cumulative incidence function showed a similar pattern (Supplementary Figure 1).

**TABLE 2:** Incidence rate of death according to low-density lipoprotein cholesterol.

	Low-density lipoprotein			
Outcomes	All levels	Low (< 2.26 mmol/L)	Moderate (2.26-2.60 mmol/L)	High (> 2.60 mmol/L)
**All-cause mortality**				
Deaths, n	820	292	181	347
Deaths, per 1,000 person-years	58.0	63.8	46.5	61.3
**Cardiovascular mortality**				
Deaths, n	415	163	88	164
Deaths, per 1,000 person-years	29.4	35.6	22.6	29.0

*The incidence rate was calculated by dividing the proportion of events by the total effective observation time in the risk, which is converted to the number of episodes per 1,000 years.*

In the multivariate cause-specific hazard model, the adjusted HRs of all-cause mortality were 1.35 (95% CI, 1.09 to 1.66) and 1.36 (95%CI, 1.13 to 1.64) for the low and high groups, respectively, compared with the moderate group, after adjustments for demographic factors, comorbidities, medication use and laboratory variables (the multivariate model in Table 3). Similarly, in the multivariate cause-specific hazard model, the adjusted HRs of cardiovascular mortality were 1.64 (95% CI, 1.22 to 2.19) and 1.31 (95%CI, 1.10 to 1.72) for the low and high groups, respectively, compared with the moderate group (the multivariate model in Table 3).

**TABLE 3:** Association between low-density lipoprotein cholesterol and all-cause mortality*.

	HR (95% CI) by low-density lipoprotein		
	Low (< 2.26 mmol/L)	Moderate (2.26-2.60 mmol/L)	High (> 2.60 mmol/L)
Univariate model	1.36 (1.13 to 1.64)	1.0	1.31 (1.09 to 1.57)
Multivariable model	1.35 (1.09 to 1.66)	1.0	1.36 (1.13 to 1.64)
Patients without prior cardiovascular disease	1.38 (1.11 to 1.72)	1.0	1.36 (1.12 to 1.66)
Patients without deaths during the first 2 year of follow-up	1.59 (1.20 to 2.10)	1.0	1.67 (1.30 to 2.15)
Patients with follow-up period ≥ 24 months	1.59 (1.20 to 2.10)	1.0	1.67 (1.30 to 2.15)
Patients with age ≥ 18 years	1.34 (1.09 to 1.66)	1.0	1.37 (1.13 to 1.65)
Patients without statin use	1.28 (1.02 to 1.60)	1.0	1.34 (1.10 to 1.63)

*Unless stated, model adjusted for age, sex, body mass index, systolic blood pressure, current smoker, current alcohol use, comorbidities, medication use, and laboratory variables. HR, hazards ratio.

We confirmed this association using a subdistribution hazard model. As compared with the reference range, higher levels of LDL-C (> 2.60 mmol/L) were associated with increased risks of all-cause mortality (HR, 1.37; 95% CI, 1.14 to 1.65) and cardiovascular mortality (HR, 1.32; 95% CI, 1.08 to 1.73). Lower levels of l LDL-C (< 2.26 mmol/L) were also associated with increased risks of all-cause mortality (HR, 1.36; 95% CI, 1.10 to 1.67) and cardiovascular mortality (HR, 1.66; 95% CI, 1.24 to 2.23) compared with the reference range (multivariate model in Supplementary Tables 1, 2).

### Sensitivity Analysis

We performed sensitivity analyses in patients without prior cardiovascular disease, without deaths during the first 2 years of follow-up, the follow-up period ≥ 24 months, or age ≥ 18 years, respectively. Similar results were observed in patients without prior cardiovascular disease, those with age ≥ 18 years, and those without statin use (Tables 3, 4 and Supplementary Tables 1, 2). Notably, higher adjusted HRs of all-cause and cardiovascular mortality were observed in patients without deaths during the first 2 years of follow-up and those with a follow-up period ≥ 24 months (Tables 3, 4 and Supplementary Tables 1, 2).

**TABLE 4:** Association between low-density lipoprotein cholesterol and cardiovascular mortality*.

	HR (95% CI) by low-density lipoprotein		
	Low (< 2.26 mmol/L)	Moderate (2.26-2.60 mmol/L)	High (> 2.60 mmol/L)
Univariate model	1.55 (1.20 to 2.01)	1.0	1.27 (1.08 to 1.64)
Multivariable model	1.64 (1.22 to 2.19)	1.0	1.31 (1.10 to 1.72)
Patients without prior cardiovascular disease	1.69 (1.24 to 2.29)	1.0	1.30 (1.08 to 1.72)
Patients without deaths during the first 2 year of follow-up	2.57 (1.70 to 3.89)	1.0	1.89 (1.28 to 2.79)
Patients with follow-up period ≥ 24 months	2.57 (1.70 to 3.89)	1.0	1.89 (1.28 to 2.79)
Patients with age ≥ 18 years	1.64 (1.22 to 2.21)	1.0	1.32 (1.11 to 1.74)
Patients without statin use	1.59 (1.12 to 2.18)	1.0	1.33 (1.10 to 1.78)

*Unless stated, model adjusted for age, sex, body mass index, systolic blood pressure, current smoker, current alcohol use, comorbidities, medication use, and laboratory variables. HR, hazards ratio.

### Subgroup Analyses

Associations of low-density lipoprotein cholesterol with all-cause and cardiovascular mortality were shown in Supplementary Tables 3, 4. We found that malnutrition (defined as serum albumin < 36.0 g/L) modified the association between LDL-C and cardiovascular mortality (*P* for interaction = 0.010, Supplementary Table 4). In further analysis, a significantly increased risk of cardiovascular mortality was observed among patients with malnutrition and lower levels of LDL-C (HR 2.96, 95%CI 1.43-6.12) or higher levels of LDL-C (HR 2.81, 95%CI 1.38-5.72). In contrast, there was no significant association among those without malnutrition. There were no other significant subgroup interactions.

## Discussion

In this real-world study of 3,565 incident Chinese CAPD patients, we found a U-shaped association between levels of LDL-C and the risk of all-cause and cardiovascular mortality, with low and high levels associated with an increased risk. The optimal range of LDL-C associated with the lowest risk of all-cause and cardiovascular mortality was 2.26 to 2.60 mmol/L (87 to 101 mg/dL). Our findings were robust because we showed consistent results across different hazard models and several sensitivity analyses. These new results in PD patients were likely to have implications for the interpretation of levels of LDL-C in clinical practice. The cut-off of the range of LDL-C 2.26-2.60 mmol/L deserved a pathogenetic hypothesis, especially focusing on the potential role of low LDL-C levels on cardiovascular mortality. As we know, high levels of LDL-C are associated with increased risks of all-cause and cardiovascular mortality. The association between low levels of LDL-C and increased risks of all-cause and cardiovascular mortality could be explained by reverse causation. Comorbidities have been reported to cause a decrease in levels of LDL-C (17) and are prevalent in dialysis patients. Thus, lower levels of LDL-C may be associated with high risks of all-cause and cardiovascular mortality. Based on our findings, keeping the appropriate range of LDL-C over a long time may improve the prognosis of CAPD patients. In addition, Early publications estimated that 40 to 66 percent of PD patients in the United States are malnourished (18–23). When evaluating the association of LDL-C with cardiovascular mortality, we should simultaneously pay attention to the nutrition status of dialysis patients, especially potential causes of hypoalbuminemia such as very low protein intake and decompensated cirrhosis.

Continuous ambulatory peritoneal dialysis patients tend to show elevated levels of total cholesterol and LDL-C and decreased levels of HDL-C (24). Contrary to the general population, lowering levels of LDL-C did not provide benefits for dialysis patients. In the 4-D study, 1255 hemodialysis patients (80 percent were not treated with a statin) with type 2 diabetes and elevated serum LDL cholesterol levels were randomly assigned to the placebo or atorvastatin group (7). After four weeks, atorvastatin successfully lowered LDL cholesterol (3.10 to 1.90 mmol/L [121 to 72 mg/dL]) versus no change with placebo (3.20 to 3.10 mmol/L [125 to 120 mg/dL]). At a median follow-up of four years, however, there was no difference in the incidence of the combined endpoint of death between both groups (HR, 0.92, 95% CI 0.77-1.10). In the AURORA trial, 2776 statin-naive hemodialysis patients were randomly assigned to the rosuvastatin (10 mg/day) or placebo group (8). At three months, mean serum LDL cholesterol levels were lowered significantly with rosuvastatin (2.60 to 1.50 mmol/L [100 to 58 mg/dL]) versus no change with placebo (2.56 to 2.53 mmol/L [99 to 98 mg/dL]). At a median follow-up period of 3.8 years, the incidence of the primary composite endpoint (death from cardiovascular causes, non-fatal myocardial infarction, or non-fatal stroke) was similar in the two groups (9.2 versus 9.5 events per 100 patient-years; HR 0.96, 95% CI 0.84-1.11). The individual components of the primary composite endpoint and all-cause mortality were also not significantly different between the two groups. Active therapy did not provide benefits for any prespecified subgroups. A meta-analysis had moderate- to high-quality evidence that, among patients on dialysis, statin treatment had little or no effect on all-cause mortality (HR 0.96, 95% CI 0.88-1.04), cardiovascular mortality (HR 0.94, 95% CI 0.82-1.07), and major cardiovascular events (HR 0.95, 95% CI 0.87-1.03) (25). Based on the findings above, the 2013 KDIGO guidelines recommend that statin therapy not be routinely initiated in dialysis patients (11). In the present study, we found a U-shaped association between levels of LDL-C and the risk of mortality in CAPD patients. Our findings are consistent with those in the general population from Denmark’s well-designed prospective cohort study (5). They reported that in the general population, the association between levels of LDL-C and the risk of all-cause mortality was U-shaped, with low and high levels associated with an increased risk of all-cause mortality. They also found that the lowest risk of all-cause mortality was at an LDL-C level of 3.60 mmol/L (140 mg/dL). In our study, the lowest risk of mortality was found at a level of LDL-C of 2.43 mmol/L (94mg/dL). Notably, the 4-D study and AURORA study lowered levels of LDL-C to 1.90 mmol/L and 1.50 mmol/L, respectively, which both were significantly lower than 2.43 mmol/L. Based on our findings, too lower levels of LDL-C were associated with increased risk of mortality. Despite focusing on different dialysis population, this may be why lowering LDL-C did not provide benefits in these two well-designed studies due to too strictly managing of LDL-C levels. Nonetheless, due to markedly difference in baseline variables and the feature of retrospective study, our findings need to be confirmed by large well-designed prospective cohort study.

Our previous study found that hyperlipidemia may harm long-term survival in diabetes mellitus patients on CAPD (26). In this study, among diabetes mellitus patients, hyperlipidemia was as a high risk of mortality as non-hyperlipidemia (HR 1.02, 95%CI 0.73 to 1.43) during the overall follow-up period, but from 48-month follow-up onwards, hyperlipidemia patients had 3.60 (95%CI 1.62 to 8.01)-fold higher risk of all-cause mortality than those non-hyperlipidemia. In the present study, sensitivity analyses found that higher adjusted HRs of all-cause and cardiovascular mortality were observed in patients without deaths during the first 2 years of follow-up and those with a follow-up period ≥ 24 months. These findings also suggested that lower or higher LDL-C levels may have a long-term adverse effect on mortality in CAPD patients. In 4-D and AURORA studies, survival plots showed that lowering LDL-C had a better long-term prognosis in hemodialysis patients (7, 8). However, the authors did not report and further analyze these findings. Further studies regarding the association between the management of serum LDL-D and prognosis in dialysis patients should have enough long follow-up period.

The strengths of our study included a large number of patients, high completeness of real-world data, and rigorous different multivariate hazard models. Nevertheless, some limitations should be mentioned. First, as a retrospective observational cohort study, this study cannot necessarily prove causation between LDL-C and mortality. A potential limitation was the possibility of residual confounding from unmeasured variables. Second, the lack of LDL-C during the follow-up period was a significant limitation, which may underestimate the association between LDL-C levels and mortality due to regression dilution bias (27). However, regression dilution bias may lead to over-adjustment (28). Third, missing values were replaced by the most recent available deals, not using multiple imputations. Although multiple imputations can randomly fill these missing values, the most recent available values may more appropriately present a patient’s clinical status. Fourth, malnutrition was only defined by serum albumin, not including prealbumin, serum cholesterol, body mass, or muscle. Lastly, all eligible patients were from China, suggesting our findings may lack generalization to other ethnic populations.

In conclusion, low and high levels of LDL-C at the start of PD procedures were associated with increased all-cause and cardiovascular mortality risks. Plus, the appropriate range of LDL-C of 2.26 to 2.60 mmol/L (87 to 101 mg/dL) was associated with the lowest mortality risk. Simultaneously, we should pay attention to the nutrition status because it may modify the association of LDL-C with cardiovascular mortality. Our findings suggested that maintaining the appropriate range of LDL-C via lipid management may improve the prognosis in CAPD patients. Nonetheless, if confirmed in more studies, our findings will have significant clinical and public health implications.

## Data Availability Statement

The raw data supporting the conclusions of this article will be made available by the authors, without undue reservation.

## Ethics Statement

The studies involving human participants were reviewed and approved by the Ethics Committee of The First Affiliated Hospital of Zhengzhou University, Zhengzhou, China. The Ethics Committee of The First Affiliated Hospital of Nanchang University, Nanchang, China. The Ethics Committee of Jiujiang No. 1 People’s Hospital, Jiujiang, China. The Ethics Committee of Zhujiang Hospital of Southern Medical University, Guangzhou, China. The Ethics Committee of The Second Affiliated Hospital of Guangzhou Medical University, Guangzhou, China. Written informed consent for participation was not provided by the participants’ legal guardians/next of kin because: The data were anonymous, and the need for informed consent was waived.

## Author Contributions

XWu: conceptualization. XWu, JW, and LZ: methodology. XWu and LZ: software. JW: validation. XWa and XZ: formal analysis and investigation. XWa, XZ, FP, YW, and XF: resources. NW: data curation. LZ: writing—original draft preparation. XWu and JW: writing—review and editing. All authors contributed to the article and approved the submitted version.

## Conflict of Interest

The authors declare that the research was conducted in the absence of any commercial or financial relationships that could be construed as a potential conflict of interest.

## Publisher’s Note

All claims expressed in this article are solely those of the authors and do not necessarily represent those of their affiliated organizations, or those of the publisher, the editors and the reviewers. Any product that may be evaluated in this article, or claim that may be made by its manufacturer, is not guaranteed or endorsed by the publisher.
