# Supplementary material for: Low-Density Lipoprotein Cholesterol and Mortality in Peritoneal Dialysis
Source: Front Nutr. 2022 Jul 21;9:910348. doi: 10.3389/fnut.2022.910348 (PMC9351358; doi:10.3389/fnut.2022.910348)
Supplement: Supplementary file 1 [file Data_Sheet_1.docx]

**Low-density lipoprotein cholesterol and mortality in peritoneal dialysis**

**Materials & Methods**

**Study Design and Participants**

**Peritoneal dialysis centers of five tertiary hospitals**

The First Affiliated Hospital of Zhengzhou University, Zhengzhou, China.

The First Affiliated Hospital of Nanchang University, Nanchang, China.

Jiujiang No. 1 People’s Hospital, Jiujiang, China.

Zhujiang Hospital of Southern Medical University, Guangzhou, China.

The Second Affiliated Hospital of Guangzhou Medical University, Guangzhou, China.

**Data Collection**

Patients were advised to initiate peritoneal dialysis (PD) with professional and clinical evaluation from nephrologists. In all patients, thorough medical records were reviewed by trained nurses in each dialysis center at study entry. In China, the patient must receive the first dialysis in the hospital, suggesting most of the patient's data can be obtained within one week before the first dialysis. Thus, we defined baseline as one week (5.3±1.2 days) before the first continuous ambulatory peritoneal dialysis (CAPD). All laboratory parameters from fasting blood samples were measured in each tertiary hospital's laboratory department.

**Dialysis procedure**

All patients received CAPD treatment. Conventional dialysis solutions (Dianeal 1.5%, 2.5%, or 4.25% dextrose; Baxter Healthcare, Guangzhou, China), Y sets, and twin bag systems were used in all CAPD patients. No patients received automated PD.

**Follow-up**

There was no exposure to all patients with any intervention. Patients needed to return to each center at least quarterly for an overall medical assessment. The trained nurses conducted monthly face-to-face interviews or monthly telephone interviews to assess their general condition and related medications.

**Outcome measurements**

We determined death causes based on medical files of admission. If patients died out of hospitals, we determined death causes according to interviewing with family members by telephone to acknowledge death's circumstances, combining with information from medical records of peritoneal dialysis centers.

**Definitions**

Cardiovascular mortality included death associated with an acute myocardial ischemic event, heart failure, hemorrhagic or thromboembolic stroke, malignant arrhythmia, and sudden cardiac death, based on the International Classification of Diseases Clinical Modification, 9th Revision. Sudden cardiac death is defined as unexpected, nontraumatic death occurring within 1hour of the onset of new or worsening symptoms (witnessed arrest) or, if unwitnessed, within 24 hours of last being seen alive[1]. Hypertension was defined as systolic blood pressure > 140 mmHg, diastolic blood pressure > 90 mmHg, or the use of antihypertensive medications. Diabetes mellitus was defined as a history of diabetes mellitus. Current smoking was defined as at least one cigarette a day, and current alcohol consumption was defined as > 20 g of ethanol a day[2]. The Chronic Kidney Disease Epidemiology Collaboration equation was used to calculate eGFR[3].

**Missing data**

In China, the patient must receive the first dialysis procedure in the hospital. To obtain missing patients' demographic characteristics, comorbid conditions, medication use, and laboratory variables, we had checked the medical records of receiving the first PD procedure. Thus, missing data for low-density lipoprotein cholesterol (n=39) or any other explanatory variables (n=147) at the start of PD were replaced by the most recent available values by checking patients’ medical records of receiving the first PD procedure. The primary outcome was not missed.

**Table S1. Association between low-density lipoprotein cholesterol and all-cause mortality using subdistribution hazard model ***

|  | HR (95% CI) by low-density lipoprotein | | | |
| --- | --- | --- | --- | --- |
|  | Low (< 2.26 mmol/L) | Moderate (2.26-2.60 mmol/L) | | High (> 2.60 mmol/L) |
| Univariate model | 1.37 (1.13 to 1.65) | | 1.0 | 1.31 (1.10 to 1.58) |
| Multivariable model | 1.36 (1.10 to 1.67) | | 1.0 | 1.37 (1.14 to 1.65) |
| Patients without prior cardiovascular disease | 1.40 (1.12 to 1.74) | | 1.0 | 1.37(1.13 to 1.67) |
| Patients without deaths during the first 2 years of follow-up | 1.48 (1.12 to 1.96) | | 1.0 | 1.58 (1.23 to 2.02) |
| Patients with follow-up period >= 24 months | 1.50 (1.14 to 1.99) | | 1.0 | 1.58 (1.24 to 2.03) |
| Patients with age >= 18 years | 1.35 (1.10 to 1.67) | | 1.0 | 1.37 (1.14 to 1.65) |
| Patients without statin use | 1.29 (1.03 to 1.62) | | 1.0 | 1.34 (1.10 to 1.64) |

*Unless stated, model adjusted for age, sex, body mass index, systolic blood pressure, current smoker, current alcohol use, comorbidities, medication use, and laboratory variables. HR, hazards ratio.

**Table S2. Association between low-density lipoprotein cholesterol and cardiovascular mortality using subdistribution hazard model ***

|  | HR (95% CI) by low-density lipoprotein | | |
| --- | --- | --- | --- |
|  | Low (< 2.26 mmol/L) | Moderate (2.26-2.60 mmol/L) | High (> 2.60 mmol/L) |
| Univariate model | 1.58 (1.22 to 2.04) | 1.0 | 1.28 (1.10 to 1.66) |
| Multivariable model | 1.66 (1.24 to 2.23) | 1.0 | 1.32 (1.08 to 1.73) |
| Patients without prior cardiovascular disease | 1.72 (1.27 to 2.33) | 1.0 | 1.31 (1.08 to 1.73) |
| Patients without deaths during the first 2 years of follow-up | 2.39 (1.58 to 3.62) | 1.0 | 1.78 (1.21 to 2.63) |
| Patients with follow-up period >= 24 months | 2.43 (1.60 to 3.67) | 1.0 | 1.79 (1.21 to 2.64) |
| Patients with age >= 18 years | 1.67 (1.24 to 2.24) | 1.0 | 1.34 (1.12 to 1.75) |
| Patients without statin use | 1.63 (1.18 to 2.23) | 1.0 | 1.34 (1.10 to 1.79) |

*Unless stated, model adjusted for age, sex, body mass index, systolic blood pressure, current smoker, current alcohol use, comorbidities, medication use, and laboratory variables. HR, hazards ratio.

**Table S3. Association of low-density lipoprotein cholesterol with all-cause mortality in subgroups.**

|  | HR (95% CI) by low-density lipoprotein | | |  |
| --- | --- | --- | --- | --- |
|  | Low (< 2.26 mmol/L) | Moderate (2.26-2.60 mmol/L) | High (> 2.60 mmol/L) | P-interaction |
| ≥65 years | 1.84 (0.91-3.69) | 1.0 | 1.96 (1.04 -3.68) | 0.476 |
| <65 years | 1.54 (1.14 -2.10) | 1.0 | 1.64 (1.25-2.16) |  |
| Male | 1.69 (1.16-2.45) | 1.0 | 1.92 (1.38-2.67) | 0.246 |
| Female | 1.52 (0.99 - 2.33) | 1.0 | 1.39 (0.95-2.04) |  |
| Diabetes mellitus | 2.09 (1.04-4.18) | 1.0 | 2.01 (1.11-3.63) | 0.324 |
| No diabetes mellitus | 1.52 (1.11-2.07) | 1.0 | 1.62 (1.23 -2.14) |  |
| Prior cardiovascular disease | 1.07 (0.39 -2.93) | 1.0 | 1.46 (0.66-3.21) | 0.893 |
| No prior cardiovascular disease | 1.68 (1.26-2.26) | 1.0 | 1.66 (1.27-2.16) |  |
| Hypertension | 2.49 (1.35-4.59) | 1.0 | 2.01 (1.11-3.64) | 0.529 |
| No hypertension | 1.66 (1.03-2.67) | 1.0 | 1.57 (1.00-2.48) |  |
| Albumin <36.0 g/L | 1.50 (1.02-2.22) | 1.0 | 1.58 (1.08-2.30) | 0.249 |
| Albumin >=36.0 g/L | 2.34 (1.43-3.86) | 1.0 | 1.85 (1.13-3.04) |  |

All analyses adjusted for age, sex, body mass index, systolic blood pressure, current smoker, current alcohol use, comorbidities, medication use, and laboratory variables. HR, hazards ratio.

**Table S4. Association of low-density lipoprotein cholesterol with cardiovascular mortality in subgroups.**

|  | HR (95% CI) by low-density lipoprotein | | |  |
| --- | --- | --- | --- | --- |
|  | Low (< 2.26 mmol/L) | Moderate (2.26-2.60 mmol/L) | High (> 2.60 mmol/L) | P-interaction |
| ≥65 years | 1.55 (0.77-3.14) | 1.0 | 0.95 (0.48-1.86) | 0.791 |
| <65 years | 1.65 (1.19-2.28) | 1.0 | 1.40 (1.04-1.88) |  |
| Male | 1.82 (1.22-2.70) | 1.0 | 1.40 (0.97-2.01) | 0.654 |
| Female | 1.45 (0.93-2.26) | 1.0 | 1.25 (0.83-1.86) |  |
| Diabetes mellitus | 2.45 (1.14-5.24) | 1.0 | 1.66 (0.84-3.31) | 0.590 |
| No diabetes mellitus | 1.53 (1.11-2.11) | 1.0 | 1.23 (0.93-1.67) |  |
| Prior cardiovascular disease | 1.14 (0.36-3.62) | 1.0 | 1.17 (0.47-12.43) | 0.633 |
| No prior cardiovascular disease | 1.69 (1.24-2.29) | 1.0 | 1.30 (0.98-2.89) |  |
| Hypertension | 1.68 (1.15-2.44) | 1.0 | 1.40 (1.00-1.95) | 0.623 |
| No hypertension | 1.59 (0.98-2.58) | 1.0 | 1.15 (0.72-1.83) |  |
| Albumin <36.0 g/L | 2.96 (1.43-6.12) | 1.0 | 2.81 (1.38-5.72) | 0.010 |
| Albumin >=36.0 g/L | 1.70 (0.96-3.02) | 1.0 | 1.12 (0.63-2.00) |  |

All analyses adjusted for age, sex, body mass index, systolic blood pressure, current smoker, current alcohol use, comorbidities, medication use, and laboratory variables. HR, hazards ratio.

**
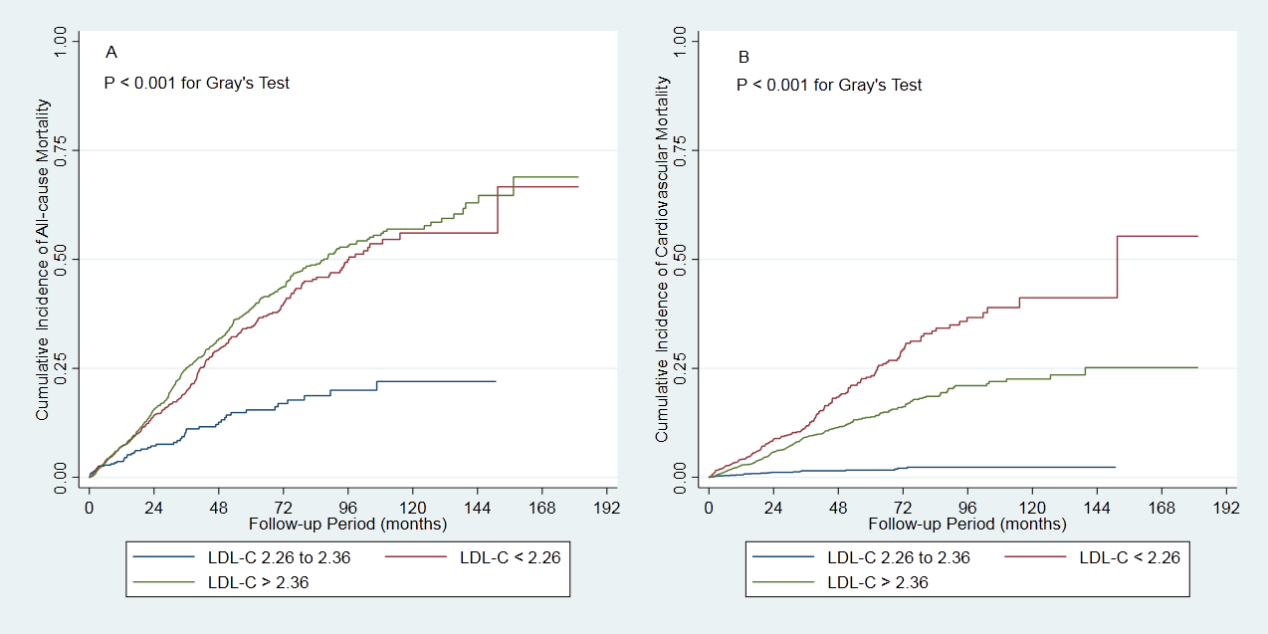
**

**Figure S1. Adjusted cumulative mortality by categories of low-density lipoprotein cholesterol.**

Panel A showed cumulative all-cause mortality by categories of LDL-C. Panel B showed cumulative cardiovascular mortality by categories of LDL-C. Adjusted for age, sex, body mass index, systolic blood pressure, current smoker, current alcohol use, comorbidities, medication use, and laboratory variables.

LDL-C, low-density lipoprotein cholesterol.

**Reference**

[1] European Heart Rhythm A, Heart Rhythm S, Zipes DP, Camm AJ, Borggrefe M, Buxton AE, et al. ACC/AHA/ESC 2006 guidelines for management of patients with ventricular arrhythmias and the prevention of sudden cardiac death: a report of the American College of Cardiology/American Heart Association Task Force and the European Society of Cardiology Committee for Practice Guidelines (Writing Committee to Develop Guidelines for Management of Patients With Ventricular Arrhythmias and the Prevention of Sudden Cardiac Death). J Am Coll Cardiol. 2006;48:e247-346.

[2] Tu W, Wu J, Jian G, Lori J, Tang Y, Cheng H, et al. Asymptomatic hyperuricemia and incident stroke in elderly Chinese patients without comorbidities. Eur J Clin Nutr. 2019;73:1392-402.

[3] Zhang L, Wang F, Wang L, Wang W, Liu B, Liu J, et al. Prevalence of chronic kidney disease in China: a cross-sectional survey. Lancet. 2012;379:815-22.
